# Quality of life and socioeconomic indicators associated with survival of myeloid leukemias in Canada

**DOI:** 10.1002/jha2.62

**Published:** 2020-07-10

**Authors:** Sonya Cressman, Donna E. Hogge, Mark D. Minden, Stephen Couban, Aly Karsan, Raewyn Broady, Emily McPherson, Khalif Halani, Jing Yi Weng, Stuart J. Peacock

**Affiliations:** ^1^ Faculty of Health Sciences Simon Fraser University Burnaby British Columbia Canada; ^2^ Department of Cancer Control Reasearch BC Cancer Research Centre Vancouver British Columbia Canada; ^3^ Canadian Centre for Applied Research in Cancer Control (ARCC) Vancouver British Columbia Canada; ^4^ Leukemia Bone Marrow Transplant Program of BC Vancouver General Hospital Vancouver British Columbia Canada; ^5^ Terry Fox Laboratories British Columbia Cancer Research Centre Vancouver British Columbia Canada; ^6^ Department of Medical Oncology and Hematology Princess Margaret Hospital Toronto Ontario Canada; ^7^ Department of Medicine Queen Elizabeth II Health Sciences Centre Halifax Nova Scotia Canada; ^8^ Centre for Clinical Genomics Michael Smith Genome Sciences Centre Vancouver British Columbia Canada; ^9^ Cancer Genetics Laboratory British Columbia Cancer Agency Vancouver British Columbia Canada; ^10^ Deptartment of Pathology and Laboratory Medicine University of British Columbia Vancouver British Columbia Canada; ^11^ Mathematica Policy Research Washington DC; ^12^ Emmes Canada Burnaby British Columbia Canada

## Abstract

Understanding how patient‐reported quality of life (QoL) and socioeconomic status (SES) relate to survival of acute myeloid leukemia (AML) and myelodysplastic syndrome (MDS) may improve prognostic information sharing. This study explores associations among QoL, SES, and survival through administration of the Euro‐QoL 5‐Dimension, 3‐level and Functional Assessment of Cancer Therapy‐Leukemia and financial impact questionnaires to 138 adult participants with newly diagnosed AML or MDS in a longitudinal, pan‐Canadian study. Cox regression and lasso variable selection models were used to explore associations among QoL, SES, and established predictors of survival. Secondary outcomes were changes in QoL, performance of the QoL instruments, and lost income. We found that higher QoL and SES were positively associated with survival. The Lasso model selected the visual analog scale of the EQ‐5D‐3L as the most important predictor among all other variables (*P* = .03; 92% selection). Patients with AML report improved QoL after treatment, despite higher mean out‐of‐pocket expenditures compared with MDS (up to $599 CDN/month for AML vs $239 for MDS; *P* = .05), greater loss of productivity‐related income (reaching $1786/month for AML vs $709 for MDS; *P* < .05), and greater caregiver effects (65% vs 35% caregiver productivity losses for AML vs MDS; *P* < .05). Our results suggest that including patient‐reported QoL and socioeconomic indicators can improve the accuracy of survival models.

## 1 INTRODUCTION

Acute myeloid leukemia (AML) and myelodysplastic syndrome (MDS) are malignancies of myeloid lineage, affecting around 10 individuals per 100 000, every year. Incidence rates nearly triple for those 70 and older and survival outcomes are poorer with increased age [1, 2]. Decisions to offer curative treatments with allogeneic stem cell transplants (alloSCT) are guided by risk factors that are a function of increased age and/or disease progression [3, 4]. Most other treatment options are, generally, noncurative in intent. The National Comprehensive Cancer Network (NCCN) guidelines consider cytogenetic abnormalities, leukemogenic mutations, co‐morbidities, and geriatric conditions with established prognostic value [5, 6, 7, 8]. Relatively less is known about the contribution of other risk factors that are independent of age or disease progression, such as quality of life (QoL) and socioeconomic status (SES). If other risk factors independently contribute to survival outcomes, then the accuracy of prognostic risk models may be improved through standardized data collection and incorporation into real‐world models.

There is a growing literature that suggests patient‐reported outcomes, such as QoL, may improve the accuracy of risk models used to predict survival outcomes. Incorporating patient‐reported fatigue, for example, into the International Prognostic Scoring System for MDS (IPSS) has improved survival prediction [9]. The prognostic value of patient‐reported health status for AML is less clear. One study in Italy reports a positive association between patient‐reported QoL scores and survival of elderly adults with AML [10], whereas in Canada, the same association has not been made [11, 12].

Missing or invisible prognostic data—such as patient‐reported QoL—may be a source of error in evaluating chances of successful treatment if it is impactful. There is reason to believe that QoL both impacts survival and is a measurable outcome for treatment. Long‐term AML survivors have reported poorer QoL compared to age‐matched members of the population without exposure to the disease or treatment [13]. The ability to evaluate new treatments also requires QoL data to estimate cost‐effectiveness [14]. Because economic models depend on data from patients, and these QoL data do not exist, the ability to evaluate new treatments would be improved with more knowledge in this area.

Socioeconomic indicators are another unexplored and potentially impactfulmissing data. Studies in the Swedish population, have shown that elderly patients with geographic access to intensive treatment for AML live longer than those without [15]. If risks such as geographic access are found to be impactful on survival, then policy to address equitable access can be developed. If inequitable access to treatment remains an issue, however, the resulting survival data that are generated will inevitably suffer from selection bias. Black people with AML in the United States, for example, have less access to curative treatment for AML, inadequate alloSCT donor availability, and poorer outcomes after treatment—therefore, they are less likely to be able to contribute long‐term survival data to predictive models and their health outcomes remain underrepresented [16]. Access to follow‐up care is further restricted by tiers of insurance coverage, thus outcomes commonly overreport results for patients who are privately insured [17].

Knowledge on the contribution that patient‐reported outcomes and/or SES has on survival and the resulting datasets generated could lead to improved modeling and better communication between clinicians and to patients and their families. In this study, we aim to explore the potential of these data to improve the accuracy of survival modeling and our understanding of how QoL and socioeconomic indicators change following treatment for AML and MDS. The study was part of the Terry Fox Research Institute's Prognostic Risk study for AML and MDS (NCT01685619), undertaken at six study centers across Canada.

## 2 MATERIALS AND METHODS

### 2.1 Patients

Adults (age > 19) with a suspected diagnosis of AML or MDS were invited to join the observational, prognostic risk study, at six major cancer centers across Canada. The study was designed to collect biological, clinical, and patient‐reported outcomes data from newly diagnosed patients with AML or MDS. The overall study objective was to explore prognostic indicators related to remission and survival including genomic and molecular indicators under investigation by the laboratory‐based investigators, and clinical outcomes such as survival, QoL, health status, and personal financial impacts for patients with AML or MDS, over 24 months of follow‐up. Patients were considered for enrollment in part two of the study if a diagnosis of AML or MDS was confirmed from a bone marrow biopsy and patients re‐consented to the study. Demographic characteristics were obtained at baseline and changes to QoL and personal financial impacts were assessed over five follow‐up time points: baseline (T0), 3 months (T1), 6 months (T2), 12 months (T3), 18 months (T4), and 24 months (T5). Recruitment started in October 2013 and ceased in March 2015 due to discontinuation in the funding for the laboratory‐based objectives, whereas funding for the clinical objectives was retained to complete follow‐up for participants who had enrolled prior to this date.

### 2.2 Cytogenetic risk factors

Cytogenetic risk for AML was assigned according to current NCCN guidelines [4], with the addition of results from molecular testing. Specifically, all participating institutions had adopted routine testing for mutations of *FLT3* and *NPM1* for intermediate‐risk AML, and *ckit* for low‐risk AML. Molecular testing was only undertaken if individuals were candidates for induction/CR1 consolidation treatment, that is, without significant age or comorbidities to preclude treatment with alloSCT. If molecular testing results for *FLT3* or *NPM1* for intermediate risk or *ckit* mutations for core binding factor t(8;21) or inv[16] AML were unavailable, an unconfirmed risk group status was assigned and included in the regression analysis as a dummy variable. All MDS‐related cytogenetic changes according to the WHO 2008 classification scheme were considered to be adverse‐risk AML.

### 2.3 Questionnaires

Following REB approval at each of the six study centers, study coordinators trained in administering the questionnaires conducted interviews in person or over the telephone, at each of the time points. The questionnaires were translated into Canadian‐French for participants in the province of Quebec. Sociodemographic characteristics, treatments, outcomes, and reasons for missing data were reported on case report forms at each of the subsequent time points and returned to a central database. The questionnaire package included the Euro‐QoL 5‐Dimension, 3‐Level (EQ‐5D‐3L) to measure QoL according to preference‐weighted health utility and Functional Assessment of Cancer Therapy‐Leukemia (FACT‐LEU) and personal financial impact questionnaires. Clinicians provided subjective measures of health status at each time point with measures of Karnofsky Performance Scores along with hospital days, survival, and remission status. Results from the EQ‐5D‐3L were scored according to preference weights specific for Canada, to generate Canadian preference‐based index utility values [18]. Individual FACT‐LEU scores were calculated using directions from the questionnaire provider [19]. The percentage of individual income devoted to healthcare expenditures was determined by dividing the sum of each participant's self‐reported expenses (specifically related to treatment), by their monthly income. Both individual and household income data were collected and the self‐reported income measures were adjusted to account for income sharing within a household. The method for determining travel expenses, loss of productivity, and caregiver impacts is provided in full detail in the Supporting Information.

### 2.4 Instrument validity and reliability

The validity of the EQ‐5D‐3L and FACT‐LEU was assessed for all participants, at each time point, through intra‐instrument item correlations and inter‐instrument total score correlations. The reliability of the FACT‐LEU was evaluated with the coefficient for Cronbach's alpha and the corrected item total correlation scores for both instruments were calculated, at each time point. Correlation with EQ‐5D‐3L index scores and the visual analogue scale (VAS) measured the relationship of the index score to an individual's own perception of QoL.

### 2.5 Endpoints and statistics

Overall survival was defined as the time from enrollment in the study to either death or 24 months of follow‐up, whichever occurred first. Event rates were the ratio for the number of events to the person years at risk for the event. The median overall survival of the cohort was estimated using Kaplan Meir life tables and log rank equality of survivor functions. Variables from established prognostic risk models (ie, age > 60, platelets, transfusion dependence, mutational status, cytogenetic risk, and missing cytogenetic results, for AML and IPSS‐R parameters for MDS) were included a priori. All other study variables with *P*‐values below .15 in a univariable analysis were included in the Cox regression and excluded by backward selection if their exclusion improved the Akaike information criteria score for the model. The coding of the variables and their selection for inclusion in the model were further refined to minimize any collinearity in the models. A complete list of all of the variables tested individually may be found in the Supporting Information. The proportional hazards assumption was tested in all models and a decision was made to adjust, stratify the model, or separately model effects for any variables that showed time‐dependent characteristics.

We used the least absolute shrinkage and selection operator (Lasso) method to enhance the accuracy and interpretation of the results from the Cox model [24]. The Lasso method allows all variables at multiple time points to compete simultaneously to add information to the model and selects the most competitive variables for inclusion. Postselection inference methods were used to compute valid *P*‐values for the Lasso model [25]. A bootstrapped sensitivity analyses was undertaken with 1000 resamples for all models and the Lasso results.

### 2.6 Multistate modeling of QoL and survival outcomes

Multistate modeling was applied to the cohort data to simultaneously analyze QoL and survival outcomes. The method involves defining a set of health states that the cohort may experience and calculating the probability of transitions between them [20]. We distinguished health states with observed or anticipated differences in mean QoL scores, mortality, relapse rates, and/or healthcare costs. The QoL data and transition probabilities (ie, the probability of moving between health states over a defined period of time) were calculated for each of the health states identified. The transition process following the initial baseline diagnosis and QoL data were modeled instantaneously and all other transitions were modeled with Weibull distributions, annually using a semi‐Markov process starting from the date of diagnosis or to either death, relapse, or follow‐up. Any post‐remission relapse/transformation was modeled from the date of relapse or transformation to death or follow‐up.

For each health state identified, the mean EQ‐5D‐3L index and FACT‐LEU scores were calculated. Any missing QoL data were accounted for by imputing missing values on health states with at least 60% of the complete data. Use of an imputed dataset is critical to analyses of patient‐reported outcomes in this disease area to adequately represent missing date due to adverse health status. The reason for missing data was recorded on the case report form and analyzed for each participant and time point. If the reason was related to poor health status (ie, not random), the imputed data were generated from the mean of the lowest quintile of the complete dataset, for each time point (T0‐T5). If the cause for the missing data was random, an imputation model was developed for each time point, using baseline and time‐dependent covariates to generate the missing values (see Supporting Information).

### 2.7 Personal financial impacts

Mann‐Whitney rank sum tests were used to test mean differences in out‐of‐pocket expenditures and productivity income losses due to time off from paid work and QoL differences related to the health states modeled for this cohort. We used Chi‐squared or Fishers exact tests to distinguish frequencies of catastrophic healthcare spending, adverse impacts on caregiver productivity, and characteristics of nonresponding participants.

## 3 RESULTS

**FIGURE 1:**
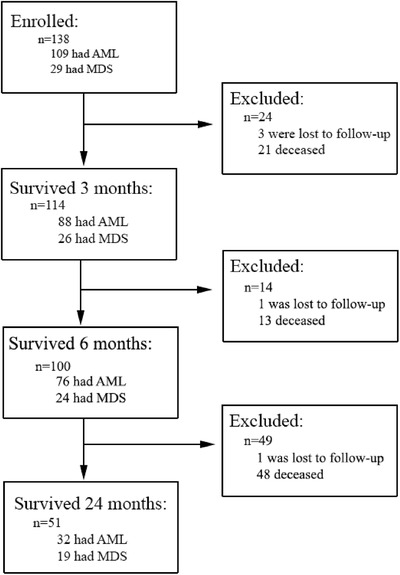
Enrolment and attrition of eligible participants over the 24‐month term of follow‐up

**FIGURE 2:**
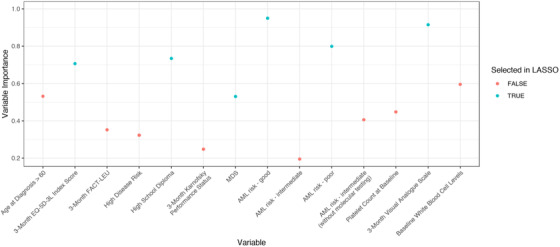
Variable importance and selection by the Lasso model. Variable importance is the proportion of bootstrap resamples in which the variable was selected

The study enrolled 188 potential participants prior to confirmation of an AML or MDS diagnosis. Diagnostic biopsies confirmed that 41 did not have AML or MDS, and nine either did not wish to provide consent or died prior to the second phase of enrollment. There were 138 participants enrolled and the cohort had a mean survival time of 463 days (95% CI, 353‐724). Of these, 51 individuals survived the 24‐month duration of follow‐up; 32 of the long‐term survivors had AML and 19 had MDS (Figure 1). The baseline demographic characteristics suggest adequate representation of individuals with lower income (38%) and other demographic characteristics (Table 1). The EQ‐5D‐3L VAS scores were the least correlated with age of all the of the QoL measures and therefore were selected for inclusion in multivariable Cox regression models (Table 2). Two models, using baseline (T0) and month three (T1) EQ‐5D‐3L VAS scores, showed that the inclusion of this variable, and an SES indicator (high‐school education or higher), improved the accuracy of prognostic information in the model, after adjustment for risk factors with known prognostic value, such as disease type, baseline platelet counts, and age above 60. The accuracy of both baseline and month three models improved when the results were stratified for males and females; however, the results from the models did not change when the sex‐stratified models were analyzed separately, with this sample size. When all variables were allowed to compete for inclusion in the Lasso model, the QoL and SES variables were always selected along with the established risk factors used for the NCCN guidelines and IPSS. The Lasso model selected the month 3 EQ‐5D‐3L VAS scores as the most important predictor (*P* = .03; 92% successful selection) (Figure 2).

**TABLE 1:** Baseline characteristics and unadjusted hazards for overall survival

		Unadjusted hazard ratios	
		
	Baseline frequency (%)N = 138^a^	HR (95% CI)	*P*‐value
Median age (IQR, Range)	64 (53‐73, 18‐91)	1.02 (1.00‐1.03)	.01
<40 years	20 (14.5%)	1 (reference)	
40‐49 years	10 (7.3%)	1.40 (0.45‐4.43)	.56
50‐59 years	24 (17.4%)	1.43 (0.56‐3.63)	.45
60‐69 years	38 (28.0%)	2.17 (0.94‐4.99)	.07
70‐79 years	33 (23.9%)	2.76 (1.18‐6.46)	.02
≥80 years	13 (9.4%)	1.61 (0.54‐4.82)	.39
Sex (% male)	71 (51%)	1.08 (0.69‐1.67)	.32
Ethnicity			
White	107 (78%)	1.33 (0.66‐2.67)	.43
Non‐White	20 (14%)		
Missing response	11 (8%)		
Marriage and cohabitation status			
Married or common‐law	88 (64%)	0.93 (0.57‐1.49)	.75
Living alone	30 (22%)	1.24 (0.74‐2.07)	.43
Net income median (IQR)^b^	$27 500 ($15 000‐$42 500)	1.00 (1.00‐1.00)	.07
Income levels			
<$20 000/year	32 (37.7%)	1 (reference)	
$20 000‐49 999	38 (44.7%)	0.62 (0.31‐1.24)	.18
≥$50 000/year	15 (17.7%)	1.47 (0.68‐3.13)	.32
Missing response	53 (38%)		
Income < $20 000/year		1.88 (1.02‐3.45)	.04
Formal Education (in seven levels)			
Less than grade 8 (level 1)	10 (7.8%)	1 (reference)	
Grades 9‐11	38 (29.5%)	0.26 (0.12‐0.58)	.00
High school diploma	18 (14.0%)	0.46 (0.20‐1.08)	.07
Some college	22 (17.1%)	0.19 (0.08‐0.49)	.00
Some university	25 (19.4%)	0.35 (0.15‐0.79)	.01
Bachelor's degree	7 (5.4%)	0.36 (0.15‐0.91)	.08
Graduate degree	9 (7.0%)	0.39 (0.15‐0.91)	.07
Missing response	9 (7.0%)		
Disease type			
AML (vs MDS)	109 (79.0%)	1.70 (0.94‐3.10)	.08
De novo AML^c^ (vs all others)	92 (68.2%)	1.08 (0.67‐1.74)	.74
AML risk group^d^			
Favorable	17 (12.3%)	1 (reference)	.00
Intermediate	19 (13.8%)	5.43 (1.53‐19.32)	.01
Adverse	48 (34.8%)	7.19 (2.20‐23.52)	.00
Missing cytogenetic results	6 (4.4%)	32.90 (6.45‐170.22)	.00
Low and intermediate IPSS‐R	20 (14.5%)	2.69 (0.71‐10.15)	.14
High and very high IPSS‐R	9 (6.5%)	4.37 (1.04‐18.34)	.04

Abbreviations: AML, acute myeloid leukemia; CR1, first complete remission; IPSS‐R, International Prognostic Scoring System for MDS, revised; IQR, interquartile range; SCT, stem cell transplant.

a The frequency of missing responses are reported if the total is greater than 5%.

b Self‐reported baseline net household or individual income, adjusted for marital status.

c Newly diagnosed AML, without known history of MDS.

d AML risk group according to 2017 National Comprehensive Cancer Network guidelines.

**TABLE 2:** Baseline and three month adjusted survival models

Variable	Baseline model (T0)		Month three model (T1)	
	HR (95% CI)	*P*‐value	HR (95% CI)	*P*‐value
Disease type^a^				
MDS	1 (reference)		1 (reference)	
Low‐risk AML	0.71 (0.19‐2.66)	.61	0.57 (0.06‐5.51)	.62
Intermediate‐risk AML	2.45 (1.17‐5.10)	.02	3.43 (1.24‐9.48)	.02
High‐risk AML	4.37 (2.07‐9.25)	<.01	5.07 (1.61‐15.99)	<.01
AML with missing cytogenetics	18.71 (3.31‐105.83)	<.01	–	–
Age above 60	2.66 (1.49‐4.74)	<.01	1.31 (0.56‐303)	.53
Baseline platelets^b^				
<50 × 10^9^/L	1 (reference)		1 (reference)	
50‐120 × 10^9^/L	0.94 (0.54‐1.66)	.84	0.99 (0.45‐2.16)	.98
>120 × 10^9^/L	1.96 (1.04‐3.68)	.04	2.00 (0.79‐5.02)	.14
First complete remission	–	–	0.40 (0.18‐0.87)	.02
Some high school or higher	0.30 (0.14‐0.66)	<.01	0.31 (0.12‐0.82)	.02
EQ‐5D‐VAS^b^	0.98 (0.97‐0.99)	<.01	0.97 (0.95‐0.99)	<.01
Sample size (n respondents)^c^	126		79	

a Integrated cytogenetic and genomic risk according to 2017 National Comprehensive Cancer Network guidelines for transplant‐eligible patients with AML

b Visual analogue scale measures from the Euroqol five‐dimension, three‐level questionnaire at either baseline (T0) or month three (T1)

c The model is stratified by male and female sex

The correlation coefficients for the QoL instruments were close to 1 when drawing comparison between the FACT‐LEU and EQ‐5D‐3L (ranging between 0.68 and 0.87), suggesting good convergent validity between the two QoL instruments. Correlation of the EQ‐5D‐3L index score with the VAS, however, indicated the instrument had poor construct validity (alpha ranged between 0.40 and 0.68) across all time points. The corrected item total correlations (CITC) indicated only fair reliability for both instruments, with some CITC scores falling below the commonly referenced thresholds for reliability of 0.2; specifically, the CITC score for the anxiety and depression item on the EQ‐5D‐3L at month 6 was 0.16 and the social and family well‐being subscale of the FACT‐LEU at baseline was 0.17.

**FIGURE 3:**
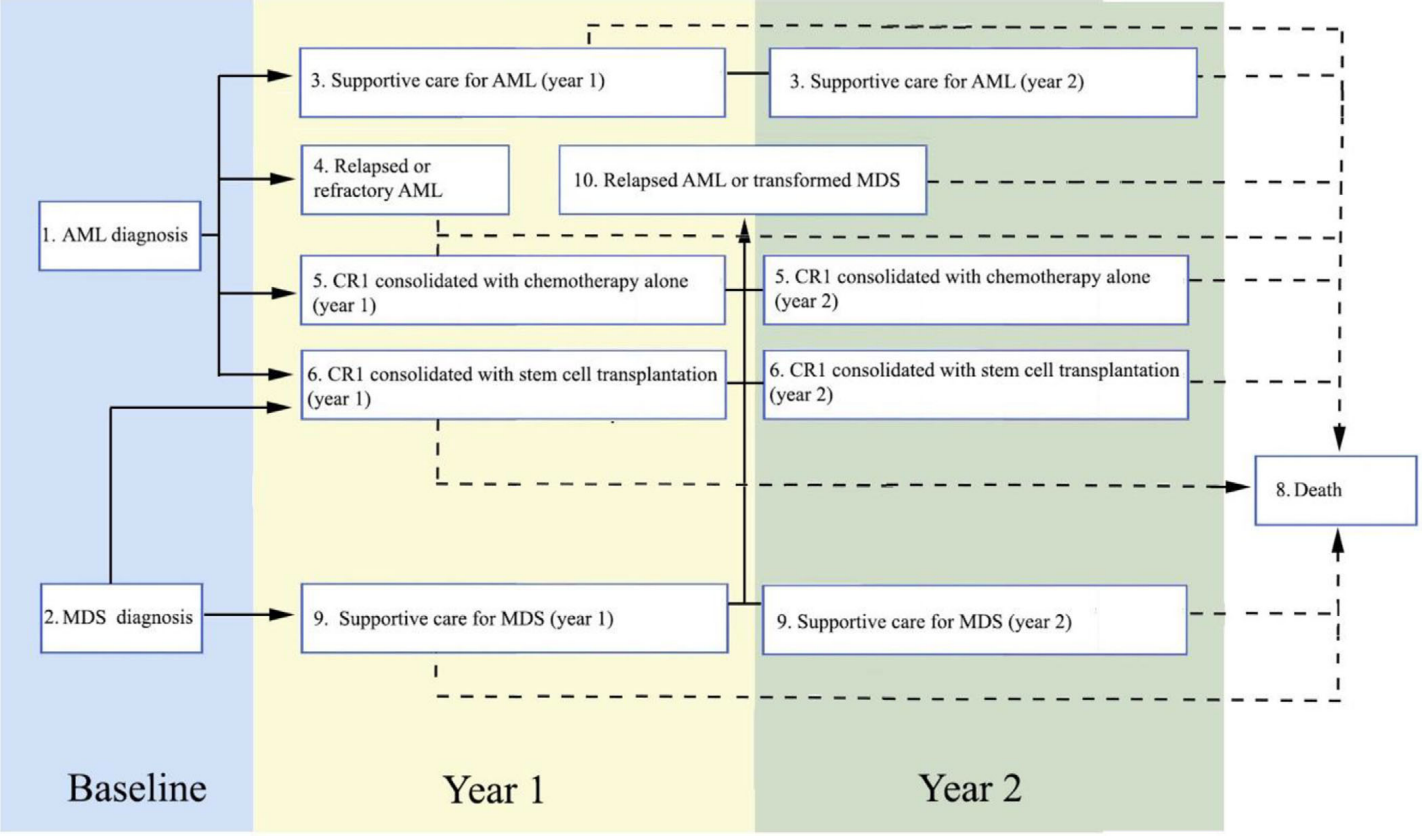
Health states definitions

The multistate modeling defined 10 finite health states for this cohort (Figure 3 and Table 3). Mean QoL scores were found to improve over time for patients with AML who had a complete remission and remained stable over time for patients who received standard care for MDS. The least preferred health states were those with the highest mortality rates, including newly diagnosed, relapsed, refractory, or transformed AML. The parameters for the parametric survival models used to calculate transition probabilities are provided in the Supporting Information. Questionnaire response rates from participants were highest for the first three study time points (greater than 65%) and fell as low as 47% for T4 due to inconsistency in study staffing, when the funding of the study was under review. Response rates returned to 65% after the study review period for T5. The majority of missing questionnaire data were missing at random due to discontinuity of staffing (see Supporting Information). A comparison of the characteristics of participants with missing responses showed that missing data were from younger participants and from one study center with the most participants and discontinuous staffing (*P* < .05). AML patients who had remissions longer than 6 months and those with MDS were more likely to respond with scores of perfect health, indicating a ceiling effect for the instrument and potential aggreability bias for people with more desirable outcomes.

**TABLE 3:** Health states, quality of life scores, and transition probabilities

Survey time point(s) and transition process modelled	Health state	Time	N(% missing^a^)	Mean EQ‐5D‐3L index score (95% CI)	Mean FACT_LEU(95% CI)	Health state transitions	Transition probability^b^(%, Standard Error)
Baseline, instantaneous transitions	1. New AML diagnosis	Baseline^c^	109 (0)	0.58 (0.53‐0.63)	99.64 (94.79‐104.49)	3. Supportive care for AML	6.42 (2.34)
						4. Refractory/relapsed AML	13.76 (3.30)
						5. CR1‐CHEMO^c^	37.61 (4.60)
						6. CR1‐SCT^c^	21.10 (3.91)
						7. Lost to follow‐up	3.67 (1.80)
						8. Death	17.43 (3.64)
	2. New MDS diagnosis	Baseline	29 (0)	0.80 (0.75‐0.86)	126.47 (119.11‐133.83)	9. Supportive care for MDS	72.41 (8.30)
						6. CR1‐SCT^c^	17.24 (7.01)
						8. Death	6.89 (4.71)
3‐24 months, semi‐Markov transitions, from date of diagnosis to relapse, death, or follow‐up	3. Supportive care for AML	Year 1	7 (29%)	0.77 (0.64‐0.90)	122.85 (101.98‐143.72)	8. Death	24.33 (16.22)
		Year 2	5 (20%)	0.76 (0.56‐0.96)	126.51 (84.92‐168.10)	8. Death	59.68 (18.54)
	4. Relapsed or refractory AML^d^	Year 1	15 (40%)	0.60 (0.44‐0.76)	110.48 (95.39‐125.58)	8. Death	81.85 (19.95)
		Year 2	5 (80%)	n.a.^a^	n.a.^a^	8. Death	99.74 (1.33)
	5. CR1‐CHEMO^c^	Year 1	41 (17%)	0.68 (0.59‐0.77)	116.65 (106.52‐126.77)	8. Death	9.04 (4.48)
						10. Relapse or transformation	20.95 (6.36)
		Year 2	28 (33%)	0.80 (0.74‐0.86)	136.89 (127.64‐146.14)	8. Death	12.15 (5.10)
						10. Relapse or transformation	38.73 (7.61)
	6. CR1‐SCT^c^	Year 1	28 (19%)	0.73 (0.66‐0.80)	119.47 (111.00‐127.94)	8. Death	14.96 (6.74)
						10. Relapse or transformation	17.26 (7.14)
		Year 2	17 (24%)	0.74 (0.66‐0.82)	132.46 (121.61‐143.31)	8. Death	26.19 (8.31)
						10. Relapse or transformation	13.73 (6.50)
	9. Supportive care for MDS	Year 1	21(19%)	0.74 (0.62‐0.86)	122.64 (110.25‐135.03)	8. Death	17.78 (8.34)
						10. Relapse or transformation	7.41 (5.72)
		Year 2	16 (27%)	0.81 (0.75‐0.87)	132.06 (117.03‐147.09)	8. Death	24.40 (9.37)
						10. Relapse or transformation	11.74 (7.02)
6‐24 months, semi‐Markov transitions, from date of relapse to date of death or follow‐up	10. Relapse or transformation to AML^d^	Year 1	22 (32%)	0.65 (0.53‐0.74)	116.46 (106.05‐126.87)	8. Death	61.5 (10.42)

a Missing data for the EQ‐5D‐3L index and FACT‐LEU were accounted for with imputation if the complete data were at least 60%.

b Survival model parameters for each transition may be found in the supplementary material.

c CR1‐CHEMO denotes complete first remission with consolidation chemotherapy alone; CR1‐SCT indicates first complete remission or treatment of high‐risk MDS that is consolidated with a stem cell transplant.

d Health state “4” corresponds to any AML that relapsed within 3 months or was refractory to induction chemotherapy; health state “10” accounts for any postremission relapse or leukemogenic transformation from MDS to AML.

When compared to MDS, the mean out‐of‐pocket expenses (ie, costs for travel to clinics, accommodations, and uninsured prescription drugs) were higher for patients with AML at month three ($559 vs $239; *P* = .05) and high medical costs may persist over longer terms of follow‐up; month six mean out‐of‐pocket expenses were $334 versus $129, *P* = .06. Nearly two thirds (67%) of all study participants reported out‐of‐pocket medical expenses that totaled more than 5% of their monthly income. Patients with AML who were in remission also reported greater productivity income losses at both T1 and T2 time points compared to all other patients. At T1 (3 months), patients with AML who had productivity income loss paid an uninsured average of $1786 (95% CI, $1293‐$2278) in lost income per month compared to $708 (95% CI, $164‐$1353) reported by patients with MDS (*P* < .05). Compared with participants with MDS, participants with AML were younger (mean age 58 vs 69 for MDS; *P* < .05) and more likely to be employed in either full or part‐time work at baseline (52% of all participants with AML vs 24% of all participants with MDS, *P* < .05). Fewer than 10% of all patients who had alloSCT—and were working at baseline—returned to work within a year. In addition, participants with AML reported greater caregiver productivity losses such as foregone employment, use of vacation time, or unpaid leave for at least 6 months compared with caregiver impacts for participants with MDS (65% vs 35% at 6 months; *P* < .05). Mean hospital days over the first 6 months of treatment were similar for patients with AML and MDS (48 vs 45 days, respectively; *P* = .70).

## 4 DISCUSSION

The results from this study suggest that data on patient‐reported QoL and SES can improve the accuracy of risk models and that scores from the EQ‐5D‐3L's visual analogue scale are the most important predictor to include in post‐remission survival models. The VAS is a subjective measure reflecting a patient's own concept of QoL. Our results suggest that including the VAS or other patient‐reported QoL measures can improve the accuracy how risk is communicated to patients. The novel finding that the VAS was the most important predictor also raises questions about how well the QoL instruments represent outcomes that are meaningful to patients.

The instruments used to measure QoL in this study were found to be only moderately reliable and the EQ‐5D‐3L notably suffered ceiling effects that often indicates agreeability bias. The EQ‐5D‐5L has been developed since this analysis in an effort to mitigate ceiling effects in other disease areas. Our findings concur with the literature suggesting that measuring QoL for AML may require more specific instruments due to the severity of the disease and the wide range of treatments and outcomes implied over time [21, 22, 23]. Our results are consistent with results from elderly patients with AML that do not report differences in QoL between intensive and nonintensive treatments with the FACT‐LEU [24]. A better understanding of the association between QoL and prognosis could improve concerns over transparency and poor communication flow between patients with AML and their attending clinicians [25]. The income and productivity losses we observe agree with the literature on adverse patient‐reported outcomes for AML in other countries and studies suggesting the need for financial support for people receiving treatment for AML [26, 27, 28].

Our study is limited by missing responses arising mostly from discontinuity in study staffing. Despite this limitation, sufficient data were captured to show socioeconomic and financial disparity in this cohort. This is a novel accomplishment for this disease area, highlighting potential for including patient‐reported outcomes in future studies. The response rates were similar to those reported from other studies of these diseases with more frequent missing values for individuals with poorer survival outcomes such as AML patients who did not receive induction therapy, or whose therapy did not result in CR1. There were, however, lower response rates for income and race/ethnicity questions than other baseline demographic reply items. Our results are therefore conservative in estimating the extent of disparity for AML patients and overrepresent outcomes for healthy individuals of higher SES.

The potential for QoL and SES variables to improve the accuracy of survival models warrants further attention. QoL outcome measures should be routinely obtained in clinical trials and the question on how meaningful those outcomes are needs to be robustly explored. Collecting information on SES from patients in research studies may improve population outcomes with information on disparity or potential gaps in unemployment insurance coverage. In Canada, any out‐of‐pocket expenditure greater than 5% of a person's net income is considered catastrophic and requires policy to protect those affected [29] The information from this study therefore suggests that Canadians with AML face more financial disparity than patients with similar conditions that requires policy attention.

## CONFLICT OF INTEREST

The authors declare no conflict of interest.

## DEDICATION TO STEPHEN COUBAN

Dr Couban, the principal investigator for the TFRI prognostic risk study, died before this publication. He was a proponent in the advancement of our understanding of patient‐relevant outcomes and was enthusiastic about sharing these results with the hematology community. His influence on the lives of patients and grace of leadership guided our collaboration.

## AUTHOR CONTRIBUTIONS

Sonya Cressman, Stephen Couban, Stuart Peacock, Donna Hogge, Mark Minden, and Aly Karsan designed the research. Sonya Cressman, Emily McPherson, Khalif Halani, and Jing Yi Weng analyzed the data. All authors participated in the interpretation of the findings and the writing of the manuscript.

## Supporting information

Supporting information
