# Supplementary material for: Quality of life and socioeconomic indicators associated with survival of myeloid leukemias in Canada
Source: EJHaem. 2020 Jul 10;1(1):69–78. doi: 10.1002/jha2.62 (PMC9175748; doi:10.1002/jha2.62)
Supplement: Supplementary file 1 — Supporting information [file JHA2-1-69-s001.docx]

Supplementary material

S.1. Variables included in the Cox regression models

Table S.1. Baseline and time-dependent variables

S. 2. Missing data

Figure S.1. A) Reasons for missing data B) and non-responses rates to questions on the FACT -LEU

S. 2. 1. Multiple imputation methodology

Table S.2. Imputation models for MAR data

Table S.3. Survival model parameters

S.3. Personal Financial Impact Questionnaires

S.3.1. Health care expenditure

S.3.2. Loss to productivity and income

S.3.3. Caregiver impacts

S.1. Variables included in the Cox regression models

Table S.1 Baseline and time-dependent variables

| Variable | Median/Frequency | HR OS (unadjusted)^1^ | P value |
| --- | --- | --- | --- |
| Less than high school education (versus all others) | 10 (7.8%) | 3.26 (1.65-6.44) | 0.001 |
| Pre-existing conditions |  |  |  |
| Median number (IQR; range) | 2 (1-3; 0-6) | 1.23 (1.05-1.43) | 0.01 |
| Type of pre-existing conditions |  |  |  |
| Autoimmune disease | 13 (9%) | 1.62 (0.84-3.15) | 0.15 |
| Cardiovascular disease | 67 (49%) | 1.28 (0.82-1.99) | 0.27 |
| Pulmonary disease | 17 (12%) | 2.09 (1.19 -3.67) | 0.01 |
| CNS or psychiatric illness | 25 (18%) | 1.59 (0.95-2.67) | 0.08 |
| Endocrine | 35 (25.9%) | 1.15 (0.70- 1.89) | 0.59 |
| Gastrointestinal | 31 (23.0%) | 1.30 (0.77-2.14) | 0.33 |
| Genitourinary | 23 (17.0%) | 1.17 (0.65-2.08) | 0.6 |
| Liver | n/a^b^ | n/a | n/a |
| Non-malignant blood disorder | 13 (9.63) | 1.82 (0.94-3.54) | 0.08 |
| Prior malignancy | 35 (25.9%) | 2.18 (1.36- 3.49) | 0.00 |
| No pre-existing conditions | 31 (22.5%) | 0.54 (0.30-0.99) | 0.05 |
| Laboratory tests |  |  |  |
| Platelets (cell X 10^9^/L) |  |  |  |
| Median (IQR; range) | 56.5 (35.5-97; 5-195) | 1.00 (1.00-1.00) | 0.09 |
| 0-49.9 | 56 (42.4%) | 0.90 (0.57-1.41) | 0.64 |
| 50-99.9 | 43 (32.3%) | 0.85 (0.52-1.39) | 0.52 |
| ≥100 | 33 (25.0%) | 1.36 (0.83-2.24) | 0.22 |
| White blood cells (cell X 10^9^/L) |  |  |  |
| Median (IQR; range) | 12.5 (3.9-40; 0.5-199.9) | 1.00 (1.00-1.01) | 0.24 |
| WBC groups |  |  |  |
| 0-9.9 | 60 (44.4%) | 0.67 (0.43-1.06) | 0.09 |
| 10-49.9 | 48 (35.6%) | 1.35 (0.86-2.12) | 0.19 |
| 50.0-99.9 | 15 (11.1%) | 1.52 (0.58-2.31) | 0.69 |
| ≥100 | 12.0 (8.9%) | 1.19 (0.57-2.47) | 0.65 |
| Blasts (cell X 10^9^/L) |  |  |  |
| Median (IQR; range) | 2.67 (0.20-14.73; 0-196.71) | 1.00 (1.00-1.01) | 0.39 |
| ≥5% diagnostic bone marrow (vs <5%) | 53 (41.1%) | 1.35 (0.86-2.13) | 0.19 |
| Missing | 10 (7%) |  |  |
| Hemoglobin concentration (g/L) |  |  |  |
| Median (IQR; range) | 2.67 (0.20-14.73; 0-196.71) | 1.00(0.99-1.01) | 0.35 |
| Hemoglobin groups |  |  |  |
| High hemoglobin (≥10 g/L) | 36 (26%) | 0.76 (0.44-1.32) | 0.32 |
| Intermediate (8.0-9.9 g/L) | 65 (47%) | 0.87 (0.56-1.35) | 0.53 |
| Low hemoglobin (<8 g/L) | 37 (27%) | 1.51 (0.94-2.41) | 0.09 |
| ANC (cell X 10^9^/L) |  | 1.00 (0.95-1.04) | 0.85 |
| Median (IQR; range) | 2.15 (0.64-7.2; 0-50) |  |  |
| ANC ≥8 | 29 (21%) | 1.25 (0.75-2.07) | 0.4 |
| ANC <8 | 105 (75%) | 0.80 (0.48-1.33) | 0.4 |
| Health status variables |  |  |  |
| Transfusion dependence (red blood cells or platelets) | 42 (31%) | 0.86 (0.54-1.37) | 0.51 |
| Median Karnofsky Performance Score (IQR; range) | 70 (60-80; 20-100) | 0.98 (0.97-0.99) | 0.00 |
| Median EQ-5D-3L Index (IQR; range) | 0.71 (0.49-0.78; -0.187-1.00) | 0.30 (0.14-0.62) | 0.00 |
| Median FACT-LEU score (IQR; range) | 106.9 (86.9-125.1; 39.6-166.0) | 0.99 (0.98-1.00) | 0.00 |
| Time-dependent variables (month 3 results) |  |  |  |
| Median Karnofsky Performance Score (IQR; range) | 70 (0.25-0.70; 10-100) | 0.98 (0.97-0.99) | 0.00 |
| Median EQ-5D-3L Index (IQR; range) | 0.74 (0.66-0.180; -0.34-1.00) | 0.30 (0.14-0.62) | 0.00 |
| Median FACT-LEU score (IQR; range) | 117.5 (99.5-133.0; 33.8-168.0) | 0.99 (0.98-1.00) | 0.00 |
| Successful CR1 (versus all others) | 42 (30%) | 0.31 (0.19-0.49) | 0.00 |

^1^Variables reported with median values were tested as continuous variables and as categorical values in the sub-group analyses

S. 2. Missing data

Following each patient’s questionnaire interview, each of the study centres reviewed item responses and queried any missing items and provided information about the reason for missing data on a case report form. The mechanisms for missing data are summarized in Figure S.1, below. Much of the missing data were explained by inconsistent study staffing during the period where the study was under funding review, therefore were considered missing at random since the missing data is not likely to be explained by quality of life. Data that were missing due to illness or an active decline (missing not at random) accounted for fewer than 10% of the overall missing data (Figure S.1.A). Item non-response rates on the FACT-LEU related to questions that could potentially make patients uncomfortable (*i.e.* related to family, hope, and/or sexual health) or were not perceived to be not applicable to patients (*i.e.* effects of chemotherapy before baseline, impact on partnerships for people who are single, Figure S.1.B).

Missing quality of life data were simulated with imputation methods only if more than 5% of the complete dataset were missing. We classified missing data as MNAR if the burden of illness, end-of-life care or any other adverse health outcome was the reason for missing data indicated on the case report form. In addition, MNAR data were identified if the physician-reported Karnofsky Performance Scores (KPS) totalled less than 20 or if the patient died within 90 days of the scheduled questionnaire. We imputed MNAR data using the average QoL scores for the lowest 10% of all KPS scores in the complete dataset. Multiple imputation was used to generate MAR data, using predictive patient characteristics to estimate EQ-5D-3L and FACT-LEU scores with regression models, as described in further detail in the supplementary material. The imputed (MNAR and MAR) dataset was used to support or challenge the conclusions from the analysis of the complete dataset.


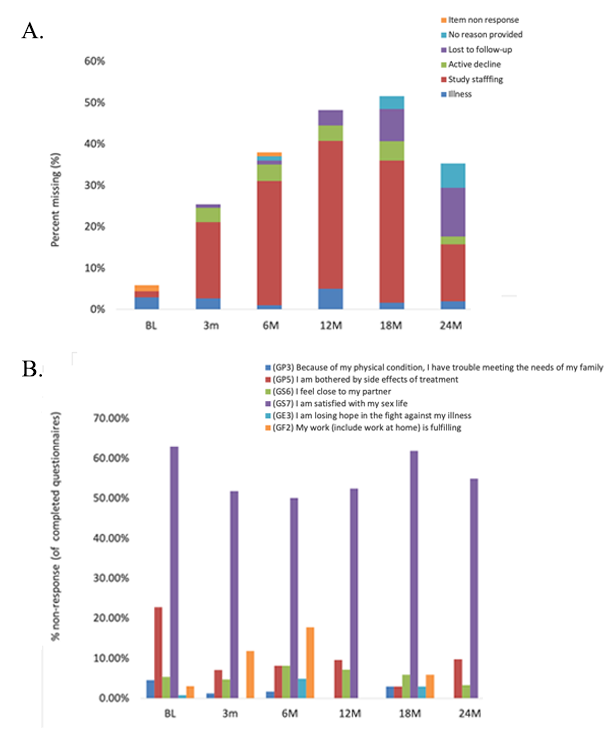


Figure S.1. A) Reasons for missing data B) and non-responses rates to questions on the FACT -LEU

S. 3. Multiple imputation

Data that were missing at random (MAR) were characterized with separate multiple imputation (MI) models built for FACT-LEU and EQ-5D index scores for each time point. The process for generating the MI models was as follows, for each instrument score and each time point. Individual regression models were built with consideration to variables that modify quality of life including time from disease diagnosis, disease type, retrospective survival time, baseline demographic socioeconomics indicators (i.e. income, education, co-habitation status, age, and treatment history for later time points including hospital stay and treatment modality/outcomes). Five candidate models were built for each timepoint and instrument. Candidate models had the highest R-squared values that were statistically significant at p<0.05. The imputation model selected for each instrument and timepoint predicted the best scores on a representative (10%, randomly selected) subgroup of the complete data.

Selection of MI model

The cumulative difference between the imputed and actual instrument scores were summed and model with the least difference was selected as the imputation model summarized in Table S.2. Five individual imputations were run and the average score from each of the five runs was used as the imputed value in the MI dataset.

Table S.2. Imputation models for data missing at random

| Time point | Instrument score | Variables used for the imputation model |
| --- | --- | --- |
| 3 month (T1) | EQ5D-3L index | EQ-5D-3L index at baseline, cohabitation (yes or no), receipt of CR1-chemo (versus all other treatments) |
|  | FACT-LEU | Cohabitation (yes or no), induction failure without salvaged remission (versus all others) baseline FACT-LEU scores |
| 6 month (T2) | EQ-5D-3L index | Cohabitation (yes or no), EQ-5D-3L month 3 score (continuous) |
|  | FACT-LEU | Chemotherapy for more than 2 months, male sex, prior hematologic condition, FACT-LEU month 3 scores (continuous) |
| 12 month (T3) | EQ-5D-3L index | Prior hematologic condition, number of hospital days in the previous year (continuous) |
|  | FACT-LEU | Relapsed AML, month 6 FACT-LEU scores |
| 18 month (T4) | EQ-5D-3L index | Number of hospital episodes, male sex and baseline smoking status |
|  | FACT-LEU | High risk MDS or AML disease status (yes or no) and age above 70 (yes or no) |
| 24 month (T5) | EQ-5D-3L index | Number of hospital episodes over two years, male sex, age above 70 |
|  | FACT-LEU | Number of hospital episodes over two years, age above 70 |

Table S.3. Survival model parameters

| Health state | Health state transition | Survival model parameters | |
| --- | --- | --- | --- |
|  |  | λ | γ |
| 1. New AML diagnosis | 3. Supportive care for AML | n/a | n/a |
|  | 4. Refractory/early relapse | n/a | n/a |
|  | 5. CR1-CHEMO | n/a | n/a |
|  | 6. CR1-SCT | n/a | n/a |
|  | 7. LTFU | n/a | n/a |
|  | 8. Death | n/a | n/a |
| 2. New MDS diagnosis | 9. Supportive care for MDS | n/a | n/a |
|  | 6. CR1-SCT | n/a | n/a |
|  | 8. Death | n/a | n/a |
| 3. Supportive care for AML | 8. Death | -13.6 | 2.1 |
| 4. Relapsed or refractory AML | 8. Death | -12.2 | 2.2 |
| 5. CR1-CHEMO | 8. Death | -9.7 | 1.2 |
|  | 11. Relapse or transformation | -11.0 | 1.6 |
| 6. CR1-SCT | 8. Death | -10.8 | 1.5 |
|  | 11. Relapse/transformation | -6.6 | 0.8 |
| 9. Usual MDS care | 8. Death | -9.2 | 1.3 |
|  | 10. Relapse/transformation | -10.8 | 1.4 |
| 10. Relapse or transformation | 8. Death | -5.8 | 1.0 |

S.3 Personal Financial Impact Questionnaires

S.3.1. Health care expenditure

The amount of personal income devoted to healthcare expenditures was determined by dividing the sum of all out-of-pocket expenses by the monthly household income reported by patients. The income was adjusted according to marital status and the mean was imputed for any missing responses to the income question on the baseline demographic questionnaire.

S.3.2. Loss of productivity

The baseline risk of productivity loss for each participant considered all contributions to productivity from full or part-time activity in employment, studies at university or college, and/or contributions to care of the home. Participants who were not on employment or received income assistance at baseline or were retired were considered not to be at risk of productivity loss. A subgroup analysis to determine return to work rates was undertaken with individuals who reported doing full or part-time work over the year prior to their diagnosis, at baseline. All subsequent impacts on work and productivity were assessed at each follow-up timepoint through questions regarding their main source of income and number of days worked over the previous month and whether or not the resumed full-time productivity (i.e. 30 hours per week of work, study, volunteering and/or care for their home per week) or part-time (1-30 hours per week of work for more than 4 weeks). Any transition from full- or part-time paid work to unpaid work, or transition from full-time work to retirement before age 65 were considered as a loss in productivity, while transition to any full or part-time employment or resumed unpaid activity compared to baseline were defined as gains in productivity.

S.3.3. Caregiver impacts

Caregiver impacts were defined as participant reported adverse impacts on caregiver’s paid work. These include the use of sick-days to provide care, cutting back on hours at work, taking unpaid leave, and foregone vacation, overtime or enrollment in courses at university specifically due to providing care for the participant with blood cancer.
